# “Is It Worth Knowing?” Focus Group Participants’ Perceived Utility of Genomic Preconception Carrier Screening

**DOI:** 10.1007/s10897-015-9851-7

**Published:** 2015-06-21

**Authors:** Jennifer L. Schneider, Katrina A. B. Goddard, James Davis, Benjamin Wilfond, Tia L. Kauffman, Jacob A. Reiss, Marian Gilmore, Patricia Himes, Frances L. Lynch, Michael C. Leo, Carmit McMullen

**Affiliations:** Center for Health Research, Kaiser Permanente Northwest, 3800 N. Interstate Ave, Portland, OR 97227 USA; Seattle Children’s Research Institute, Treuman Katz Center for Pediatric Bioethics, Seattle, WA USA; Northwest Permanente, Kaiser Permanente Northwest, Portland, OR USA

**Keywords:** Genomic preconception carrier screening, Decision-making, Whole genome sequencing, Focus groups, Barriers and facilitators, Patient perspectives, Qualitative research

## Abstract

As genome sequencing technology advances, research is needed to guide decision-making about what results can or should be offered to patients in different clinical settings. We conducted three focus groups with individuals who had prior preconception genetic testing experience to explore perceived advantages and disadvantages of genome sequencing for preconception carrier screening, compared to usual care. Using a discussion guide, a trained qualitative moderator facilitated the audio-recorded focus groups. Sixteen individuals participated. Thematic analysis of transcripts started with a grounded approach and subsequently focused on participants’ perceptions of the value of genetic information. Analysis uncovered two orientations toward genomic preconception carrier screening: “certain” individuals desiring all possible screening information; and “hesitant” individuals who were more cautious about its value. Participants revealed valuable information about barriers to screening: fear/anxiety about results; concerns about the method of returning results; concerns about screening necessity; and concerns about partner participation. All participants recommended offering choice to patients to enhance the value of screening and reduce barriers. Overall, two groups of likely users of genome sequencing for preconception carrier screening demonstrated different perceptions of the advantages or disadvantages of screening, suggesting tailored approaches to education, consent, and counseling may be warranted with each group.

**Electronic supplementary material:**

The online version of this article (doi:10.1007/s10897-015-9851-7) contains supplementary material, which is available to authorized users.

## Introduction

Compared to existing carrier screening technologies, genome sequencing offers substantially more information for reproductive decision-making. Mendelian recessive or x-linked conditions, despite being individually rare, impact as many as 2 % of live births. These conditions have an important impact on the health of affected children and account for about 20 % of infant mortality and about 10 % of pediatric hospitalizations (Costa et al. 1985; Kumar et al. 2001; Berry et al. 1987). Numerous conditions could be considered for carrier screening, with more than 1000 known autosomal recessive or x-linked conditions in the Online Mendelian Inheritance in Man (OMIM) database. This substantial genetic heterogeneity means that a large proportion of the genome must be evaluated to assess carrier status across all possible conditions. However, current clinical practice is generally limited to evaluating a small number of genes. Carrier screening is more widespread for conditions like Cystic Fibrosis (CF), which is recommended to be offered to all couples considering pregnancy in the U.S. (Grody et al. 2001; American College of Obstetricians and Gynecologists Committee on Genetics 2011), beta-thalassemia, for which population screening programs have been implemented in countries with a higher prevalence such as Greece, Italy, and Cyprus (Cao et al. 2002; Cousens et al. 2010), or Tay Sachs disease in the Ashkenazi Jewish population (Kaback 2001). However, pre-conception carrier testing is mostly offered only in specific situations that confer an increased risk. It is typically offered to family members of an identified carrier or affected individual, and members of ethnic or racial groups known to have a higher prevalence of a condition (ACOG Committee on Genetics 2009; American College of Obstetricians and Gynecologists Committee on Genetics 2011).

Commercial screening panels that incorporate tens or hundreds of disorders have recently become available, but they are usually limited to well-established gene variants. The yield of carrier findings per person from current carrier testing panels varies. One study found that 24 % of individuals were carriers of at least one of 400 causal Mendelian variants (Lazarin et al. 2013). Another study suggests that each person is a carrier of 2 to 3 mutations for known disorders (Bell et al. 2011). At the genome scale, we may expect a relatively high yield of findings per person because we can interrogate the majority of the exome or genome (Biesecker and Green 2014). Additionally, genome sequencing can provide information on incidental findings—conditions that would not impact a future child’s health, but that impact the patient directly such as mutations in *BRCA1/2* that increase risk for hereditary breast and ovarian cancer (Green et al. 2013; Kaphingst et al. 2015; Regier et al. 2015; Bennette et al. 2013). Thus, carrier screening using genome sequencing (either whole genome or whole exome, which we will refer to as “genomic carrier screening”) could identify more mutations than panel screening and, as a result, identify more couples at risk of bearing children affected by genetic conditions. Preconception carrier testing, which represents a high proportion of the care delivered in medical genetics, is a prime candidate for the use of genome sequencing. Further, carrier status is a potentially relevant secondary finding whenever genome sequencing is used in adults of reproductive age, regardless of why the sequencing was ordered. Despite the potential utility of genomic carrier screening, little is known about how patients react to findings from such tests, and whether they find such testing acceptable, understandable, and useful to their family planning.

Such data are important because genomic carrier screening lies at the intersection of two clinical activities that each raise ethical concerns. Carrier testing raises questions about whether screening programs that are designed to decease the incidence of genetic diseases will pressure adults to accept testing that they really do not want, and if such programs send messages that are harmful to the disease communities for which testing is targeted (Wilfond and Thomson 2000). Carrier testing after conception is controversial because of strongly held beliefs regarding whether it is ethical to terminate a pregnancy. Genomic sequencing raises questions about adverse impacts on people who receive information that may be difficult to interpret, which can be confusing, and may result in unnecessary clinical interventions (Wade et al. 2013; Wilfond and Goddard 2015). Obtaining data about the experience of patients can help address the veracity of these concerns, and aid in developing approaches to address them. To improve our understanding of the utility of genomic carrier screening, we explored patients’ perceptions of its advantages and disadvantages of this service (as compared to usual clinical care) through a series of focus groups. This research was conducted as part of the National Human Genome Research Institute (NHGRI) Clinical Sequencing Exploratory Research (CSER) consortium. As part of this consortium, we are currently conducting a research study on the implementation of genome sequencing for couples seeking preconception carrier testing.

## Methods

### Study Site and Background

Kaiser Permanente Northwest (KPNW) is an integrated health care delivery system that serves approximately 500,000 health plan members in the Portland, Oregon metropolitan area. The single most common genetic or genomic application used in practice today at KPNW is carrier testing for CF, which is routinely offered to women who become pregnant or are planning a pregnancy, with about 3900 tests per year. About 14 % of CF tests are for couples seeking pre-conception testing. Women who receive pre-conception carrier screening tend to be older than the average for pregnant women (34 years vs. 25 years), and are likely to receive CF testing as part of an encounter to address questions about infertility.

### Sampling and Recruitment

As part of a clinical trial of genomic preconception carrier screening at KPNW, we conducted three focus groups (Morgan 1998) to understand what factors hinder or facilitate such screening. We used data extracted from the electronic medical record (EMR) to identify women (ages 18–50) with current health plan membership, and who had preconception carrier testing in the past 5 years. A recruitment list was generated and sorted by zip code to identify 100 participants living near each of the three focus group locations. We attempted to increase the diversity of perspectives and experiences represented in the focus groups in several ways. First, sessions were intentionally held in clinics located within different socio-demographic constituencies (inner urban; suburban; and mixed urban/suburban). Second, we oversampled minority group members to compensate for limited population diversity in the metropolitan area by utilizing minority identifiers in the EMR (African–American and Asian) and concentrated initial calls for members in target zip codes.

### Data Collection

A discussion guide was iteratively developed by the research team with a final version approved by the project’s lead qualitative researcher (CM). The guide consisted of overarching questions, presented in an open-ended fashion, followed by prompts for specific feedback on certain issues (Online Resource 1). The focus group leader (CM) provided an orientation to genome sequencing and carrier screening at the beginning of each session through a brief slide presentation (Online Resource 2). Slides defined genomic carrier screening, standard carrier testing, and incidental findings (receiving results about oneself), and explained how genome sequencing differs from what has been offered traditionally in the health plan. The discussion also entailed careful review of a draft consent form for genomic carrier screening; many terms and concepts were clarified in that discussion. Additionally, a content expert—either a medical geneticist (JR) or a genetics researcher (KG), was present at each session to help clarify concepts and answer questions from participants. Discussion topics included: prior experience with genetic testing; reasons participants would/would not choose to have genomic carrier screening; awareness/understanding about the risks and benefits of screening; reactions to receiving incidental findings (IF); and preferences for returning results. All sessions were audio-recorded for verbatim transcription. Participants were consented, and all focus group procedures and materials were approved by the Institutional Review Board at KPNW.

### Data Analysis

Qualitative data analysis was aided by the use of ATLAS.ti 5.0 software (Scientific Software Development 1997) for coding data and generating reports of coded text for analysis. We engaged in a two-stage thematic analysis, starting with a grounded, open-coding approach (Strauss and Corbin 2008), followed by a more focused analysis based on participants’ perceptions of the value of genetic information. For the grounded approach, we first developed a coding dictionary based on the guide, review of the transcripts, and discussion with the qualitative study team (CM, BW, JR, TK, KG, JS, and JD). Transcribed interviews were coded by trained staff (JS), by marking passages of text with phrases (e.g. codes) indicating content. Using the query functions of ATLAS.ti, reports of coded text were generated for review and further analysis, divided both by codes (e.g., barriers to screening) and focus group session (e.g., session one).

During this review process, we noticed that more than half of the participants across the focus groups were high information seekers, while others were less so. We determined it would be revealing to distill differences among strong information-seekers and those who were more cautious about the information they wanted to receive, so that we could better understand the range of barriers and facilitators to obtaining preconception genomic carrier screening. Thus for the second stage of analysis, each transcript was carefully re-reviewed, and participants were identified as either being “certain” (e.g., made clear statements about wanting to know/seek out this information) or “hesitant” (e.g., made clear statements about feeling unsure they would want to know/seek out this information). Participants distinctly fell into one of these two orientations, with none being ambiguous based on their comments. Thematic code reports were re-generated and reviewed for each of these two orientations. We applied the same approach when reviewing focus group discussions about receiving IFs. While taking this analytical approach, we continued to review coding reports through an ongoing, inductive process, resulting in refined themes (Patton 2002; Wolcott 1994; Bernard and Ryan 2010). A trained and experienced expert in qualitative analysis who did not conduct the focus groups (JS) led the analysis process, with input and guidance from the focus group moderator and research team. This allowed for both an outsider (JS) and insider (remaining listed authors) review of the focus group data, as refined themes were continually shared with the research team and clinical staff in an ongoing process until the group reached consensus on interpretation.

## Results

### Recruitment Results and Descriptions of Participants

We made 287 recruitment call attempts, and reached 109 (38 %) individuals. Of those, 81 (74 %) declined participation (e.g., busy/unavailable = 61; not interested =12). The remaining 28 individuals were booked into the three focus groups; 12 did not show up. Overall, 16 individuals participated across the three sessions. Table 1 describes their characteristics. Focus group 1 (FG1) had six participants, five female and one male, with a mean age of 30. The male attendee was an un-recruited spouse of a participant who came to the group (consented and participated). Focus group 2 (FG2) had six female participants with a mean age of 31, and focus group 3 (FG3) had four female participants with a mean age of 28. A majority of participants described having many years of membership with the health plan, with most participants (*n* = 7) reporting somewhere between 5 years and 20 years, and several (*n* = 5) indicating a “lifetime” of 21 years or more. A few respondents described being newer to the health plan, having four or less membership years. Despite our efforts to invite minority participants, all participants were Caucasian.

**Table 1:** Characteristics of focus group participants (*n* = 16)

Focus Group (FG) Session	Gender		Age		Years as Health-plan member			
	F	M	Mean	Range	< or = 4 years	5–10 years	11–20 years	21+ years
FG 1 (*n* = 6)	5	1^a^	30	21–37	0	1	2	3
FG 2^b^ (*n* = 6)	6	0	31	28–37	2	1	1	1
FG 3 (*n* = 4)	4	0	28	25–31	1	2	0	1

All participants were Caucasian

^a^Male attendee in FG1 was un-recruited spouse of a participant who came to group, was consented and participated

^b^one participant in FG2 came in later so we did not obtain information on age or years at KPNW

### Prior History and Experience with Genetic Testing

As described above, we intentionally recruited women who appeared to have some prior preconception genetic testing experience within the health plan. During the beginning of the session, we asked participants to share their “story” of how and why they obtained prior preconception testing to help develop rapport during the session and provide a frame of reference for the discussion about genomic carrier screening in our current study. The majority of participants (*n* = 12) indicated the conversation about obtaining testing was initiated by either their primary care provider or obstetrician. Participants attributed the conversation originating from their provider often due to the participant’s age, current health status, prior fertility challenges, or health/family history.

I was talking with the nurse practitioner in the OB/GYN clinic—I had had a miscarriage about a year before. And so she recommended it. So my husband and I made the decision, since we had the miscarriage, and we wondered if there was something causing that, that had to do with our genetics.

Three participants described initiating the conversation about preconception testing with their provider, actively seeking out this information as part of their family planning approach. For most focus group participants (*n* = 10), the testing occurred before the member became pregnant in an effort to help with pregnancy planning and fertility challenges. However, about one-third (*n* = 5) described the testing occurring during their pregnancy due to their own or their partners’ health history, current health status, or family heritage. Participants made reference to receiving testing for such things as Down Syndrome, Cystic Fibrosis, and Tay-Sachs disease.

I am fifteen weeks pregnant, and this is my first pregnancy. And my husband’s sister had a Down’s pregnancy that they terminated. I don’t know for sure, but I don’t think if it weren’t for that I would have opted for genetic screening… I figure it was a good thing to do. And really, the reason we did was because that would affect our decision as far as moving forward.

Due to time constraints within the focus group session and a desire to focus the majority of the discussion on reactions to genomic carrier screening, we did not systematically explore the outcome of these prior preconception genetic testing experiences, nor did participants volunteer this information. We did, however, ask participants to reflect on any barriers or concerns with their prior preconception testing experience. The most frequently described concern was how the testing added another layer of stress to an already anxiety provoking experience of trying to become pregnant or a first time mother (*n* = 6). This is exemplified in an exchange among participants in one of the focus groups.

P#2: I feel like everything about impending motherhood is anxiety provoking. [non-verbal: All agree with head nodding or saying “yeah” over each other]…

P#4: Especially when you’re waiting for those [genetic screening] results. What are they going to say?

Other concerns expressed less frequently included: having a non-supportive or non-participatory partner in the testing process (*n* = 2); being uncomfortable with anything perceived as invasive (*n* = 2); having a negative reaction to some of the genetic testing terminology, such as the word “mutation” (*n* = 2); expense due to health plan not covering desired tests (*n* = 1); and feeling overloaded with information (*n* = 1).

### Is Genomic Carrier Screening Worth Knowing?

Following the brief discussion about participants’ prior genetic testing experience, the moderator described the current study about genomic carrier screening, how this differs from the usual clinical care participants had experienced previously (Online Resource 2), and solicited participants’ reactions. Our analysis of the ensuing discussion centered on an overarching theme that divided focus group participants’ orientation towards genomic carrier screening: Is it “worth knowing” the results of such tests? We found that participants could be characterized as having two basic orientations toward the value of this screening: those “certain” about obtaining screening (*n* = 10) represented across all three focus group sessions, and those more “hesitant” (*n* = 6), represented by three participants each in FG1 and FG2. Review of comments made by “certain” and “hesitant” participants revealed differing descriptive characteristics of these two groups as highlighted in Table 2, along with illustrative quotes.

**Table 2:** Is genomic carrier screening worth knowing? Differences between “certain” and “hesitant” participants (*n* = 16)

Certain (n = 10) (FG1 = 3; FG2 = 3; FG3 = 4) Worth knowing as knowledge is power	Hesitant (n = 6) (FG1 = 3; FG2 = 3; FG3 = 0) Not sure worth knowing as knowledge fosters uncertainty & stress
**Reasons worth knowing**	**Reasons may not be worth knowing**
• Information and knowledge gives a sense of control, lessens anxiety and helps with decision-making *Illustrative quote:* “I want the information. I want the facts, because I can deal with things better if I have the facts… I would feel more in control, in a situation where you could feel completely out of control.”	• Information won’t change choice to start a family (e.g. have children) *Illustrative quote:* “For us, it wouldn’t be serious enough that we would choose an abortion. So therefore it doesn’t really matter what the test would say.”
• May help parent engage in prevention activities and change how approach health for self and family (*diet, exercise, lifestyle & health choices*) *Illustrative quote:* “I’d want to know too. I mean maybe it will be a motivation to eat more healthfully and exercise a bit more, take supplements… [Chuckles] I mean, we all should. But I would definitely want to know.”	• Do not want to invest time, money, resources and emotions in something that may not happen *Illustrative quote:* “I’m like what is the value in this, not just for me but like bigger picture? And I don’t know that I want to invest that much time and money and other resources into more than I need… is it worth knowing?”
• May help parent prepare for the possibility of the condition (*support/conversations with partner; terminate pregnancy or not; plan as best can for future possibilities*) *Illustrative quote:* “He and I would want to know…even if I couldn’t prevent it from happening…so we could be as prepared to deal with it as possible.”	• Stress of obtaining screening may not be worth knowing just because science or healthcare system can offer it *Illustrative quote*: “What if it [genomic carrier testing] turns out positive, you still don’t know for certain…I just think it’s very stress inducing. Your partner has to get tested. And even then, your child still might not get this disease. So you might be doing all of this testing and, again, all this stress and your child could be perfectly healthy.”
• May be a cure or treatment in the future so important to know information so can be researching/looking for these options *Illustrative quote:* “I like to research… I just want to know and be prepared for the possibilities. So for me, knowing and being able to investigate more about specific areas that I could be dealing with…”	• Feels like it interferes with the “sacred” experience of pregnancy and becoming a parent / prefer more holistic approaches *Illustrative quote:* “I’m really conflicted, because pregnancy and child rearing is a timeless thing. And it’s become this medical thing… I don’t think that it [genomic carrier testing] should become the norm.”
• Late adult onset conditions may be equally important to learn about in case cure or treatments arise over the years *Illustrative quote:* I would want to know [late adult onset conditions] so that when my child is ready to have that conversation, you know, that’s when there will be cures for these things…I want you to know so that I can be listening to the research that says, ‘we’ve just found a cure to [such and such]’…I could prepare my child to be on the lookout.”	• Late adult onset conditions seem less important or necessary to know about (*too far out in future; information could be wrong; science could change*) *Illustrative quote:* “So if you tell me that my kid could have something when he’s sixty, chances are that something is going to happen that could very drastically change the course of medicine in the next sixty years. So I think it [knowing late adult onset conditions] becomes irrelevant.”

Participants who were certain considered genomic carrier screening “worth knowing” because for them, “knowledge is power.” They described how information derived from the screening could reduce anxiety and provide a sense of control that would enhance family planning decisions. They claimed that test results could help them prepare for the possibility that their offspring may have a genetic condition, including taking such actions as: engaging in supportive discussions with their partner, health care providers, or others; changing how they approach their health and lifestyle choices (e.g. nutrition, exercise) for themselves and their family; researching the condition and possible future treatments/cures; and possibly considering termination of a pregnancy. Additionally, these focus group members typically desired to know all possible results from genome sequencing, including adult onset conditions. This desire to have all possible results was driven by the belief that “knowing” the information now may assist in searching for potential cures or treatments that could arise in future years.

In contrast, participants who were hesitant about the value of genomic carrier screening were guided primarily by the belief that too much knowledge can foster uncertainty and stress. Reasons included: it would not likely change their choice or decision to start a family; it would be a waste of time, money, resources or emotions over something that may not happen; screening just because science or the health care system allows for it may not outweigh the stress of obtaining the information; and it interferes with the “sacred” and natural experience of becoming pregnant or being a parent. These participants also were more likely to want a more limited set of result categories if they did decide to have genomic carrier screening. For example, they did not think it was important or necessary to learn about later adult onset results because the potential impact of this knowledge would be too far out in the future, and the scientific knowledge about such conditions and their treatment could change by the time any affected offspring reached adulthood.

### Barriers to Seeking out Genomic Carrier Screening

Participants described four main barriers, with different orientations toward these barriers among the certain and hesitant participants, summarized in Table 3, along with illustrative quotes. The most commonly cited barrier (*n* = 9) was **fear and anxiety **about what may be discovered. The hesitant participants stated that actively choosing to not know something can be easier than coping with the stress of knowing it, especially when that information is perceived as uncertain, undesirable, or scary. They were also afraid that knowledge of screening results could interfere with life plans. Thus, actively choosing to not obtain genomic carrier screening was described as creating a sense of freedom to proceed with family planning goals unencumbered by the burden of knowing potentially difficult information about future offspring. Alternatively, the certain participants expressed fear and anxiety about findings being incomplete or not clear enough to guide family planning or care decisions for future offspring. Additionally, certain participants were anxious that they may have insufficient education and knowledge to fully understand their reproductive and family planning choices and options once they learned about results.

**Table 3:** Potential barriers to preconception genomic carrier screening: Themes and differences between “certain” and “hesitant” participants (*n* = 16)

Certain (*n* = 10) (FG1 = 3; FG2 = 3; FG3 = 4)	Hesitant (*n* = 6) (FG1 = 3; FG2 = 3; FG3 = 0)
**Fear and anxiety of what will find out**	
• Fear/anxiety that not enough certain or detailed information will be found out to guide decisions (incomplete findings)	• Coping with the fear and anxiety of genome-scale carrier testing results that could be difficult or emotional is much harder than coping with not ever having this knowledge
• Concern lack of own knowledge about what genomic carrier screening is may lead to not fully understanding choices and options *Illustrative quote: “*Going into this, I’m pretty young and pretty new to this particular world. I want as much information as possible, because with this [testing] that we’re talking about there’s a lot that can go wrong. And as parents, or somebody who wants to be a parent, you want to be as informed as possible.”	• Fear that knowledge from genomic carrier screening may interfere with family planning dreams and goals *Illustrative quote:* “If you don’t know, it’s a lot easier to go along in ignorance, and just let whatever happens happen…I think fear is a big motivator to either do something or to not do something.”
**Concerns of how and when results are shared**	
• Not being given all of the result information at once (e.g. in one visit) fosters stress/resistance to doing genomic carrier screening *Illustrative quote:* “And then in getting this testing [you] actually do a whole lot of waiting… So waiting and then [potentially] having multiple appointments… I can see how that could make you upset or even more stressed out.”	• Waiting a long time for results fosters fear, anxiety and possible resistance to doing genomic carrier screening • Potentially being treated as a ‘number’ or ‘percent’ rather than a human being creates resistance to screening *Illustrative quote:* “I’m thinking about having a baby. It could be two to three months before I know these results… And I could see for me, that causing a lot of anxiety - all of a sudden I have to start postponing things.”
**Uncertainty whether a clear need or rationale exists for testing**	
• Fascination and/or trust in science/technology and what it can provide overshadows concerns as to whether a clear need exists or not to seek out genomic carrier screening *Illustrative quote:* “As far as I know, I don’t have anything in my family that would be a cause for alarm for…and I love studies for any genetic research.”	• If no personal medical history or family history on either side of partner/family then likely would choose not to do genomic carrier screening • Put trust in higher power/ less trust in medical technology or interference *Illustrative quote:* “I wouldn’t do it unless I had some history of something I was specifically wanting to know more and feel more confident about…But for me, it’s too much information… It’d drive me crazy. I’d be worried about it too much.”
**Lack of partner participation**	
• Resistance to engage in genomic carrier screening if have incomplete data due to lack of partner involvement *Illustrative quote:* “I would be concerned how I interpreted the results if you haven’t [tested] my husband …you need to think about those X-chromosome types of conditions.”	• May create tension within partnership if lack of mutual agreement exists on whether to engage in genomic carrier screening *Illustrative quote:* “And why would you want that person to go through it alone? Because what if one has reservations and the other doesn’t? Does one unilaterally get to decide, okay, I’m doing this? And then, if you don’t agree you don’t have the do the second part. But then that creates a potential fight later on [when female results come in]…”

The second most commonly mentioned barrier (*n* = 7) related to **how genomic carrier screening results are shared**. The certain participants wanted to receive all of the result information, possibly within one (rather than multiple) visits, and were not comfortable with the fact that researchers and/or clinicians might have more information than what would be revealed to patients (such as non-actionable findings, or variants of unknown significance). Hesitant participants were concerned about the perceived “bleak” discussion of possible outcomes that would be part of the consent process for such screening or the terminology used to convey such information. Hesitant participants demonstrated more sensitivity to the language and terminology used in describing genomic carrier screening, reacting negatively to terms like “mutation,” “death,” or “medically involved.” Hesitant participants described how terms like these can foster a significant emotional reaction of fear or anger, setting one up to potentially misunderstand genomic carrier screening options and/or results. They also objected to waiting a long time for their genome sequencing results. Within the context of our study, for which participants were reacting the return of results was described to be approximately 12 weeks. This wait-time may be less in other contexts.

Two other barriers were cited by about one-third (*n* = 5) of the participants. One barrier was **lack of a clear need or rationale **for screening. Hesitant participants stated that they would likely not obtain it if they or their partner had no personal medical or family history that would increase their risk for having offspring affected by a genetic condition. Additionally, some of these participants cited they would prefer to put their trust in a “higher power” rather than in science or interference by medical technology. On the other hand, certain participants were curious about and trusting in what science and medical technology can provide. Another barrier was **lack of partner participation**. Hesitant participants mentioned concerns that lack of mutual agreement to be screened could foster tension within their partner relationship, while certain participants were more concerned with having incomplete data or results to base decisions on due to lack of partner involvement.

### Factors that Facilitate Genomic Carrier Screening

While much of the discussion focused on barriers to or concerns about obtaining genomic carrier screening, participants also shared factors that would motivate them to obtain screening. The primary motivator for the majority of participants was a desire to do “everything possible” to protect the health of their offspring. This was strongly expressed by both certain and hesitant participants. They saw value in obtaining the screening so they would know what to expect and be better prepared for themselves and their offspring. Also, they felt having this information before conception could help explain or decrease pregnancy related complications and possibly prevent miscarriages.

Because my biggest concern would be, if I decide I don’t want to find out about it, but the child has it and I don’t know, and then I do something wrong that causes a problem… So I would want to know, at least somehow to be able to help and provide that child the best care possible.

Certain and hesitant participants revealed some subtle differences in their motivation for screening. Certain participants discussed the benefit of being both financially and emotionally prepared early on, allowing them to be more capable to offer support and coping assistance to help their partner and affected offspring. Hesitant participants were more motivated to obtain testing as they believe the results could help them create a “normal life” for their affected offspring and allow the child to be a part of society in some of the same ways as children who do not have the genetic condition.

### Choices are Essential

A key recommendation strongly expressed by all participants during the discussions was the importance of providing choice at every level of offering genomic carrier screening—including choice in whether to seek out this information, the type and quantity of results being shared, how and when to receive the results, and from whom they would want to receive the results and/or have follow up conversations. Both certain and hesitant participants described how offering choice is paramount to making genomic carrier screening “worth knowing” since people will bring their unique range of emotions, expectations, and prior experiences into their decision-making. Participants described how offering clear patient choice at multiple levels can make one more open to obtaining and knowing results from genomic carrier screening, and facilitates greater acceptance of receiving potentially difficult information. Participants saw providing choice as a means to decrease fear and anxiety about obtaining screening or learning results because choice fosters a sense of control about what and how much one may want to know. As one participant summarized:

I think as much as you can, cater to each individual’s needs…as many options as you can give them about finding out about results or how to communicate with providers about it. Just be really open to everyone’s unique needs in terms of how they arrive at deciding to get genetic testing.

## Discussion

Preconception genomic carrier screening may strengthen reproductive autonomy and informed decision-making compared with prenatal screening or even existing preconception panel tests because it maximizes the number of conditions tested and the reproductive options available to couples at higher risk of bearing affected offspring (Modra et al. 2010; Borry et al. 2011). However, several opinions exist within the field about whether or how to deliver such screening, including which genome sequencing results should be returned to patients (Burke et al. 2001; Ravitsky and Wilfond 2006). With the rapid advances in genome sequencing technology and the increasing commercial availability of genomic tests, additional research is needed to guide decision-making about what types of information can or should be offered to patients, and to elucidate how patients understand and prioritize this information (Bollinger et al. 2012; Hosli et al. 2008).

We explored barriers, facilitators, and the perceived utility of preconception genomic carrier screening in focus groups with individuals of child-bearing age with prior genetic testing experience. Our analysis revealed two types of likely orientations toward preconception genomic carrier screening—certain individuals who desire all the information possible from genomic carrier screening because this knowledge provides a sense of control in their family planning decisions; and hesitant individuals who are more cautious as the knowledge obtained could possibly be anxiety-provoking, would not likely change their family planning decisions, and potentially “medicalizes” the natural experience of pregnancy and motherhood. Analysis revealed that these two types of orientations have slightly different perspectives on four participant-identified barriers to screening: fear and anxiety about results; concerns about the method of returning results; concerns about the necessity for screening; and concerns about male partner participation in screening. All participants overwhelmingly recommended offering choice to patients as a way to enhance the value of and reduce barriers to screening. Allowing patients and their partners to choose what they want to find out, and how, may allay concerns and enhance benefits of screening among both certain and hesitant individuals. These focus group findings may help address the broader ethical and clinical question regarding the utility of providing this type of screening (Burke et al. 2010; Burke et al. 2001; Remennick 2006), helping to negotiate a balance between potential benefits and harms.

Our focus group findings support and expand upon recommendations found in the literature. The American College of Medical Genetics and Genomics (ACMG) recommends prenatal/preconception expanded carrier screening processes that uphold patient choice, autonomy, and lack of harm (Grody et al. 2013). Another qualitative study about carrier screening for fragile X syndrome (FXS) also found the “right to choose” testing and results to be a unifying theme across stakeholder groups (providers, pregnant women, and general public) (Archibald et al. 2013). Our focus group participants echoed these sentiments. In describing negative aspects of their previous experience with genetic testing as stressful, unnecessarily invasive, or causing conflict with their partners, it seems that the context of such testing, namely health care utilization associated with trying to become pregnant, frames women’s orientation to preconception genetic testing, whether it is traditional or genome scale testing.

By analyzing barriers and facilitators to genomic carrier screening from the perspectives of certain/hesitant orientations, we demonstrate variability in the perceived value of such information that is also found in other studies. An exploration of informed decision-making for carrier screening for FXS in non-pregnant women (Metcalfe et al. 2008) found that the most common reason to decline FXS testing was concern about knowing “worrisome” information that would likely not alter reproductive plans; this was similar to what we found among our hesitant participants. In the case of screening for CF, research suggests that the perceived value of information shapes decisions about testing. Prenatal and preconception carrier screening for CF was introduced into routine obstetric practice in 2001 (Grody et al. 2001). There has been variable uptake: some of the variability is related to women’s values and interest in the information.

Studies of FXS and CF have described decision-making processes relating to preconception carrier screening, something we did not explore in our focus groups. Participants in our focus groups were discussing hypothetical situations, not making actual screening decisions, limiting our understanding of decision making related to genomic carrier screening. In the case of FXS, women who chose to test or not revealed a two-step decision-making process, with the first phase focusing on issues of relevancy (e.g. reproductive stage of life); followed by a stage of deliberation in which one may be influenced by factors such as valuing the knowledge from testing, perception of risk, and logistical issues (Archibald et al. 2009). In the case of CF, decision-making has been shown to vary by the context of testing (Grody et al. 2001; Ioannou et al. 2014) —whether it is presented prenatally, recommended by a provider, or requires the woman to actively seek it out (Modra et al. 2010). Future research, including our ongoing trial of preconception carrier screening, should explore whether decision making processes for genomic carrier screening resemble other carrier testing or screening scenarios, in which relevancy and the timing of decisions play an important role.

Ultimately, tailored approaches to offering genomic carrier screening could be developed to address differences in patients’ certainty regarding the utility of such screening. The differences in how certain and hesitant participants talked about barriers indicates that educational materials, consent documents, genetic counseling and primary care provider talking points could address and/or be tailored to these different orientations. Understanding the differences between certain and hesitant individuals can provide guidance in both creating materials and in training providers in how to best offer and communicate about genomic carrier screening.

Finally, we looked at comments of hesitant and certain participants regarding incidental findings and did not find clear differences between the two groups. This may mean that a person’s perceptions about the utility of preconception genomic carrier screening may not be generalizable to other types of genomic screening. Distinguishing the differences in barriers and facilitators for genetic screening when it provides information about one’s own health versus the health of one’s offspring is an area for future exploration.

### Limitations

Our study has some limitations. The small sample size and lack of minority participants may limit the use of our findings as possible guidance for others to consider. Our relatively low recruitment rate may have resulted in some selection bias among focus group participants, with more representation from participants who were enthusiastic about genomic preconception screening. Within the dynamics of a focus group discussion, some participants may not always express their opinion when it differs from others; and others may dominate discussions with their style of communication. There is a possibility that the opinions of those categorized as hesitant may be understated because they were a minority of the participants, while the certain participants may have been more vocally dominant. Additionally, those categorized as hesitant were present in only two of the focus groups. However, we employed several strategies to improve the credibility and trustworthiness of our data (Patton 2002; Morgan 1998; Denzin and Lincoln 2011), including using a trained focus group facilitator experienced in managing group dynamics; employing an interview guide to assure consistency of data collection across focus group sessions; using a formal, team-based approach to analysis that included transcription, coding, and multiple reviews of summarized themes; and reviewing our findings with project and health plan staff to get a sense of the “face” validity of our data as it compares to what they hear from patients.

## Conclusion

Advances in genome sequencing technology create great potential for informing and improving reproductive decision making; however, in making this screening available we need to understand both clinical utility and ethical considerations from the viewpoints of multiple stakeholders. This paper looks at patients’ perspectives through a series of focus groups. We found two distinct types of potential users of preconception genomic carrier screening who demonstrated differences in perceived advantages or disadvantages of screening. Our findings suggest that tailored approaches to education, consent and counseling may be warranted with each group, and may help foster informed decision-making about whether to obtain testing. Given that our focus groups members were reacting to a hypothetical scenario and were not participants within our current clinical trial, our results are most indicative of patients’ stance toward preconception genomic screening before the consent process, which involves learning about the potential benefits and harms of testing. In that context, participants in the certain group displayed what could be described as over-confidence about the value of genomic information. This over-confidence warrants clear and consistent communication about the limits of the technology, current scientific knowledge, and some of the potential downsides of testing. Hesitant participants shared the need for materials and talking points to be sensitive to terminology that is potentially confusing or anxiety-provoking; a desire to potentially avoid results pertaining to severe/life threatening outcomes or conditions impacting infancy/early childhood; suggestions to avoid being overwhelmed with “too much information” by sharing pre-consent and potential result information over multiple visits rather than just one visit; and a need for coordination of care across multiple providers (Ob/Gyn; PCP; genetic counselor) that is consistently and clearly documented within the medical chart so that the responsibility of any follow-up actions does not solely fall upon the patient, who may feel emotionally burdened by the knowledge of screening or related results.

Within our current clinical trial of preconception genomic carrier screening, we are continuing to gain knowledge about how to tailor services so that testing can be delivered in a way that responds to patient values and orientation toward genomic information. We are also collecting information about why patients decline to participate in our trial. These data will result in concrete suggestions about tailoring information and services at different phases of clinical care, including offering services, informed-decision making about testing, return of results, and guidance about future steps after obtaining results.

## Electronic supplementary material

Online Resource 1 (PDF 257 kb)

Online Resource 2 (PDF 259 kb)
