# Supplementary material for: “Is It Worth Knowing?” Focus Group Participants’ Perceived Utility of Genomic Preconception Carrier Screening
Source: J Genet Couns. 2015 Jun 21;25:135–45. doi: 10.1007/s10897-015-9851-7 (PMC4726717; doi:10.1007/s10897-015-9851-7)
Supplement: Supplementary file 1 — (PDF 257 kb) [file 10897_2015_9851_MOESM1_ESM.pdf]

Article title: *"Is it worth knowing?" Focus group participants' perceived utility of genomic preconception carrier screening*

Journal name: Journal of Genetic Counseling

Authors: Jennifer L. Schneider[1]; Katrina A.B. Goddard[1]; James Davis[1]; Benjamin Wilfond[2]; Tia L. Kauffman[1]; Jacob A. Reiss[1]; Marian Gilmore[3]; Patricia Himes[3]; Frances L. Lynch[1]; Michael C. Leo[1]; and Carmit McMullen[1].

Author's primary affiliations:

[1] Center for Health Research, Kaiser Permanente Northwest, Portland, OR

[2] Seattle Children's Research Institute, Treuman Katz Center for Pediatric Bioethics, Seattle, WA

[3] Northwest Permanente, Kaiser Permanente Northwest, Portland, OR

Corresponding Author e-mail: Jennifer.L.Schneider@kpchr.org

Caption: Online Resource 1: Focus Group Guide (Abbreviated Version)

Online Resource 1: Focus Group Guide (Abbreviated Version)

1. Based on your prior experience, what type of information was most important for your decision about whether or not to have a genetic test?
2. Our study will provide people with information about “preconception genomic carrier screening.” [Brief explanation here]. What would be some of the reasons why you would want to obtain this screening? What sort of actions might you take with this sort of information?
3. What would be some of the reasons NOT to obtain preconception genomic carrier screening?
4. We will be asking participants in this study whether they want results for many different kinds of genetic conditions or only for those that are the most severe. [Discuss categories of results] What do you think about these categories? How would you decide <u>which types</u> of results you would want to have for preconception genomic carrier screening?
5. If you found out that you were a carrier in one of these types of tests, what information would you want your genetic counselor to provide to you for preconception genomic carrier results?
6. We will also be asking participants in the study if they want to learn about genetic test results that are relevant to their own health rather than about genetic conditions that they could potentially pass along to children. [Explanation comparing preconception genomic carrier screening and incidental findings.] How would you decide <u>whether</u> you would want to learn about these types of results for incidental findings?
7. What information would you want your genetic counselor to provide to you for incidental findings?
8. What actions or choices do you think you might make if you learned you yourself had a condition due to incidental findings. [E.g., tell family, make lifestyle, related healthcare decisions]
9. What other sources of information would be helpful to you? [Other patients? Online resources? Written materials?]
10. I’d like each of you to pick one thing you want us to be sure and pay attention to when we go over what you have said in the group today. What would that be? Please take a moment and think about it, and we will go around the room one by one. Do you have any final questions? / Are there any final thoughts you would like to share?
