# Supplementary material for: “Is It Worth Knowing?” Focus Group Participants’ Perceived Utility of Genomic Preconception Carrier Screening
Source: J Genet Couns. 2015 Jun 21;25:135–45. doi: 10.1007/s10897-015-9851-7 (PMC4726717; doi:10.1007/s10897-015-9851-7)
Supplement: Supplementary file 2 — (PDF 259 kb) [file 10897_2015_9851_MOESM2_ESM.pdf]

Article title: *"Is it worth knowing?" Focus group participants' perceived utility of genomic preconception carrier screening*

Journal name: Journal of Genetic Counseling

Authors: Jennifer L. Schneider[1]; Katrina A.B. Goddard[1]; James Davis[1]; Benjamin Wilfond[2]; Tia L. Kauffman[1]; Jacob A. Reiss[1]; Marian Gilmore[3]; Patricia Himes[3]; Frances L. Lynch[1]; Michael C. Leo[1]; and Carmit McMullen[1].

Author's primary affiliations:

[1] Center for Health Research, Kaiser Permanente Northwest, Portland, OR

[2] Seattle Children's Research Institute, Treuman Katz Center for Pediatric Bioethics, Seattle, WA

[3] Northwest Permanente, Kaiser Permanente Northwest, Portland, OR

Corresponding Author e-mail: Jennifer.L.Schneider@kpchr.org

Caption: Online Resource 2: Terms /background information shared during focus group discussions

Online Resource 2: Terms /background information shared during focus group discussions	
**Differences between Incidental Findings and Preconception Genomic Carrier Screening**	
**Incidental Finding Results**	**Genomic Carrier Screening**
• Relate to *your* risk of developing a condition	• Looks for changes in your genetic material (DNA) that can cause the conditions you are being tested for<br>• Genetic carriers do not show symptoms of an inherited condition, but can pass the gene changes to their children
**Differences between Traditional and Expanded, Genomic Carrier Screening**	
**Traditional Carrier Screening (Usual care in clinic)**	**Expanded, Genomic Carrier Screening (Whole genome-sequencing)**
• Looks at one gene or a small number of genes that can lead to inherited conditions that you could pass to your children	• Looks at more genes<br>• Looks at more changes than just the most common ones<br>• Looks at genes that may be important for your health, and also genes involved in inherited conditions
• Find out about only one or a few conditions	• Information about 100 conditions that could affect your future children, and 150 conditions that could affect you<br>• Does not test for all conditions that exist
